# Sulforaphane induced cell cycle arrest in the G2/M phase via the blockade of cyclin B1/CDC2 in human ovarian cancer cells

**DOI:** 10.1186/1757-2215-6-41

**Published:** 2013-06-26

**Authors:** Chi-Chang Chang, Chao-Ming Hung, Yun-Ru Yang, Mon-Juan Lee, Yi-Chiang Hsu

**Affiliations:** 1 Department of Obstetrics & Gynecology, Tainan Sin-Lau Hospital, Tainan, Taiwan; 2 Department of Obstetrics & Gynecology, E-Da Hospital/I-Shou University, Kaohsiung, Taiwan; 3 Department of General Surgery, E-Da Hospital, I-Shou University, Kaohsiung, Taiwan; 4 Department of Bioscience Technology, College of Health Sciences, Chang Jung Christian University, Tainan, Taiwan; 5 Graduate Institute of Medical Science, College of Health Sciences, Chang Jung Christian University, Tainan, Taiwan; 6 Innovative Research Center of Medicine, College of Health Sciences, Chang Jung Christian University, Tainan, Taiwan

**Keywords:** Ovarian cancer, Sulforaphane (SFN), Cell cycle, Cyclin B1/CDC2

## Abstract

**Background:**

Malignant tumors are the single most common cause of death and the mortality rate of ovarian cancer is the highest among gynecological disorders. The excision of benign tumors is generally followed by complete recovery; however, the activity of cancer cells often results in rapid proliferation even after the tumor has been excised completely. Thus, clinical treatment must be supplemented by auxiliary chemotherapy or radiotherapy. Sulforaphane (SFN) is an extract from the mustard family recognized for its anti-oxidation abilities, phase 2 enzyme induction, and anti-tumor activity.

**Methods:**

This study investigated the cell cycle arrest in G2/M by SFN and the expression of cyclin B1, Cdc2, and the cyclin B1/CDC2 complex in PA-1 cells using western blotting and co-IP western blotting.

**Results:**

This study investigated the anticancer effects of dietary isothiocyanate SFN on ovarian cancer, using cancer cells line PA-1. SFN-treated cells accumulated in metaphase by CDC2 down-regulation and dissociation of the cyclin B1/CDC2 complex.

**Conclusion:**

Our findings suggest that, in addition to the known effects on cancer prevention, SFN may also provide antitumor activity in established ovarian cancer.

## Background

Isothiocyanates (ITCs) are naturally occurring components of vegetables that have demonstrated biological activity against carcinogenesis as well as chemopreventive properties [1]. It has been suggested that in conjunction with chemotherapy, ITCs may enhance drug sensitivity [2]. Sulforaphane (SFN), a potent cancer preventive agent, is a dietary isothiocyanate found as a precursor glucosinolate in cruciferous vegetables such as Brussels sprouts, cauliflower and broccoli [3]. Interest in this agent has grown in recent years due to its putative beneficial pharmacological effects as an antioxidant [4], anti-inflammatory [5] and antitumor agent [6]. SFN is also a potent scavenger of reactive oxygen species (ROS), including superoxide anions and hydroxyl radicals [7]. Many studies have indicated an inverse correlation between the consumption of cruciferous vegetables and a decrease in the incidence of various tumors, including those of the prostate [8], cervical [9], colorectal [10], and lung [11]. In addition to inhibiting cell proliferation and increasing apoptosis [10], other mechanisms have also been proposed to explain the anti-carcinogenic effects of SFN. These include anti-inflammatory and antioxidative activities, the induction of phase 2 detoxification enzymes, the inhibition of cyclooxygenase 2 (COX-2) [12], and the effect on protein kinases [13].

This study investigated the influence of SFN on ovarian cancer cell lines (PA-1) with regard to the anti-proliferation of PA-1 cells and induced cell cycle arrest in the G2/M phase. These results may provide support for the chemoprevention of ovarian cancer.

## Methods

### Materials

Sulforaphane [1-isothiocyanato-(4*R*,S)-(methylsulfinyl)butane], DMSO (dimethyl sulfoxide) and MTT [3-(4,5-dimethylthiazol-2-yl)-2,5-diphenyltetrazolium bromide], were obtained from Sigma (St Louis, MO). All other reagents and compounds were analytical grade.

### Cell culture

PA-1 cells were purchased from the Food Industry Research and Development Institute (Hsinchu, Taiwan). The cells were maintained in flasks containing MEM supplemented with 10% (v/v) FBS and cultured in an incubator at 37°C with an atmosphere containing 5% CO_2_.

### Cell proliferation assay

Cells were seeded into 96-well culture plates at 10,000 cells/well and treated with SFN. One to three days (0 μM SFN was the control group.) MTT dye (1 mg/ml) was added to each well 4 hours following treatment. The reaction was stopped by the addition of DMSO, and OD_540_ was measured using a multi-well plate reader (Powerwave XS, Biotek). In the absence of cells, the background absorbance of the medium was subtracted. Results were expressed as a percentage of the control, which was considered to be 100%. Each assay was performed in triplicate and the results were expressed as the mean (+/−SEM).

### Measurement of apoptosis

PA-1 cells were first seeded in 6-well plates (Orange Scientific, E.U.). Following treatment with SFN for four hours, the supernatant was discarded and cells were harvested and re-centrifuged. Cells were subsequently resuspended/incubated in 1X annexin-binding buffer (5 μL of annexin V-FITC [BD Pharmingen, BD, USA] and 1 μL of 100 μg/mL PI working solution) for 15 minutes. Following incubation, the stained cells were analyzed using flow cytometry (FACSCalibur, BD, USA). Data was analyzed using WinMDI 2.8 free software (BD, USA).

### Cell cycle analysis

Cell cycle analysis was performed using fluorescent nucleic acid dye and propidium iodide (PI) to identify the proportion of cells in each of the three stages of interphase. Cells were treated with SFN for 24 hours, and subsequently harvested and fixed in 1 ml of cold 70% ethanol for at least eight hours at −20°C. DNA was stained in PI/RNaseA solution and the DNA content was detected using flow cytometry. Data was analyzed using WinMDI 2.8 free software (BD, USA).

### Western blot assay

A total of 30–50 μg of proteins were separated by 10% SDS-PAGE and transferred to PVDF membranes (Millipore, USA). The membranes were blocked with blocking buffer (Odyddey, USA) overnight and subsequently incubated with anti-β-actin (Sigma-Aldrich, St. Louis, MO, USA), anti-CDC2, anti-caspase 3, and anti-cyclin B1 (Santa Cruz BioTechnology, USA) antibodies for 1.5 ~ 2 hours. The blots were then washed and incubated with a second antibody (IRDye Li-COR, USA) at a dilution of 1/20,000 for 30 minutes. Finally, the antigen was visualized using a near infrared imaging system (Odyssey LI-COR, USA) and data was analyzed using Odyssey 2.1 software.

### Co-immunoprecipitation (Co-IP)

Co-IP is an effective means of quantifying protein-protein interaction in cells. Briefly, 500 μg of cellular proteins were labeled using anti-cyclin B1 (Santa Cruz BioTechnology, USA) following overnight incubation at room temperature. The protein-antibody immunoprecipitates were collected by protein A/G plus-agarose (Santa Cruz BioTechnology, USA). Following the final wash, the samples were boiled and centrifuged to pellet the agarose beads. Western blot analysis of the CDC2 protein in the supernatant was then conducted. Antigens were visualized using a near infrared imaging system (Odyssey LI-COR, USA) and data was analyzed using Odyssey 2.1 software.

### Statistical analysis

All data was reported as the mean (±SEM) of at least three separate experiments. A *t*-test or one-way ANOVA with post-hoc test was employed for statistical analysis, with significant differences determined as *P* < 0.05.

## Results

### SFN inhibits proliferation of PA-1 cells

**Figure 1:**
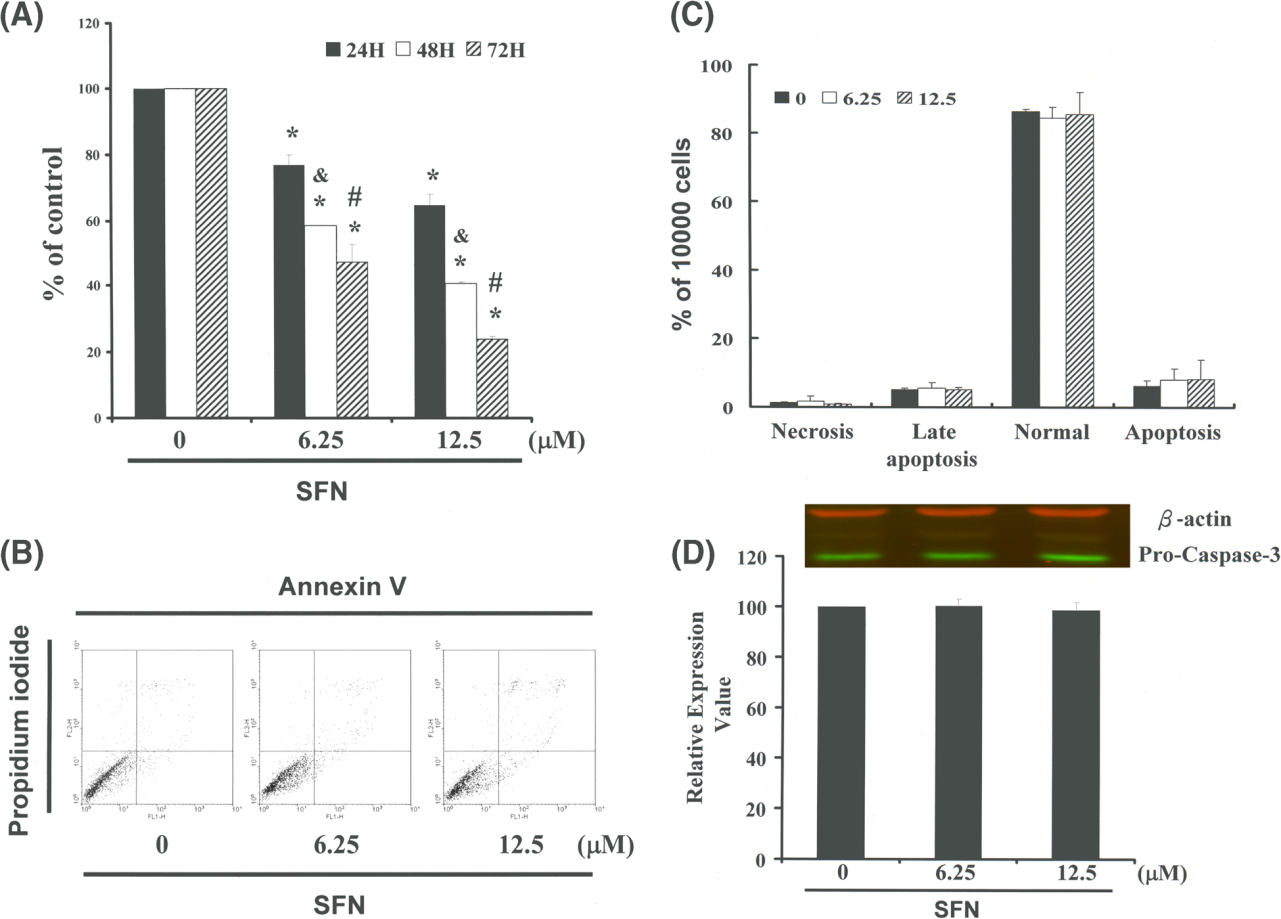
**SFN mediates the survival of PA-1 cells, and thus inhibits proliferation: (A) In vitro study was initiated by treating each of the PA-1 cells with increasing doses of SFN for 24 to 72 hours.** The survival of these SFN-treated cancer cells was then measured using the MTT method; **(B)** Influence of SFN on apoptosis in PA-1 cells; **(C)** Total apoptosis in PA-1 cells after 4 h of incubation with SFN; **(D)** Caspase-3 activation in PA-1 cells following SFN treatment. After being treated with SFN, cells underwent western blot analysis and band intensities (pro-caspase 3) were quantified using an Li-COR near infrared imaging system. Statistical analysis was performed using a *t*-test; the symbols *, & and # in each group of bars indicate that the differences resulting from treatment with SFN are statistically significant at P < 0.05.

We hypothesized that SFN could mediate the survival of PA-1 cells and thus inhibit their growth. To explore the anti-tumor activity of SFN against PA-1 cells, an in vitro cell viability study was conducted in which each sample of the PA-1 cells was treated with increasing doses of SFN (0, 6.25, and 12.5 μM) for 24 to 72 hours. The results in (Figure 1A) indicate that the survival and proliferation of PA-1 cells were decreased by treatment with SFN in a dose-dependent (The 24 hours’ IC_50_ of simvastatin in the PA-1 cancer cells was determined to be 17.083 μM; y = −2.8213× + 98.199, R^2^ = 0.9697) (*p < 0.05 vs SFN 0 μM group) and time-course manner (& p < 0.05 vs 24 hour treatment, # p < 0.05 vs 48 hour treatment).

### SFN repressed the cell viability of PA-1 cells without apoptosis induction

A study on apoptosis was performed to further elucidate anti-cancer mechanisms of SFN in PA-1 cells. After treating the cells with various doses of SFN, the percentage of apoptotic cells was assessed using Annexin V-FITC and propidium iodide staining, followed by flow cytometric analysis (Figure 1B). A dot-plot of Annexin V-FITC fluorescence versus PI fluorescence also indicated a non-significant increase in the percentage of apoptotic cells treated with SFN. At SFN concentrations of 6.25 to 12.5 μM, no significant increase was observed in the percentage of cells undergoing necrosis and apoptosis (Figure 1C) or caspase 3 activation (Figure 1D). The results summarized in Figure 1 indicate that SFN may mediate the survival of PA-1 cells and thus inhibit their proliferation without apoptosis induction.

### SFN treatment induced the accumulation of G_2_/M phase in PA-1 cells

**Figure 2:**
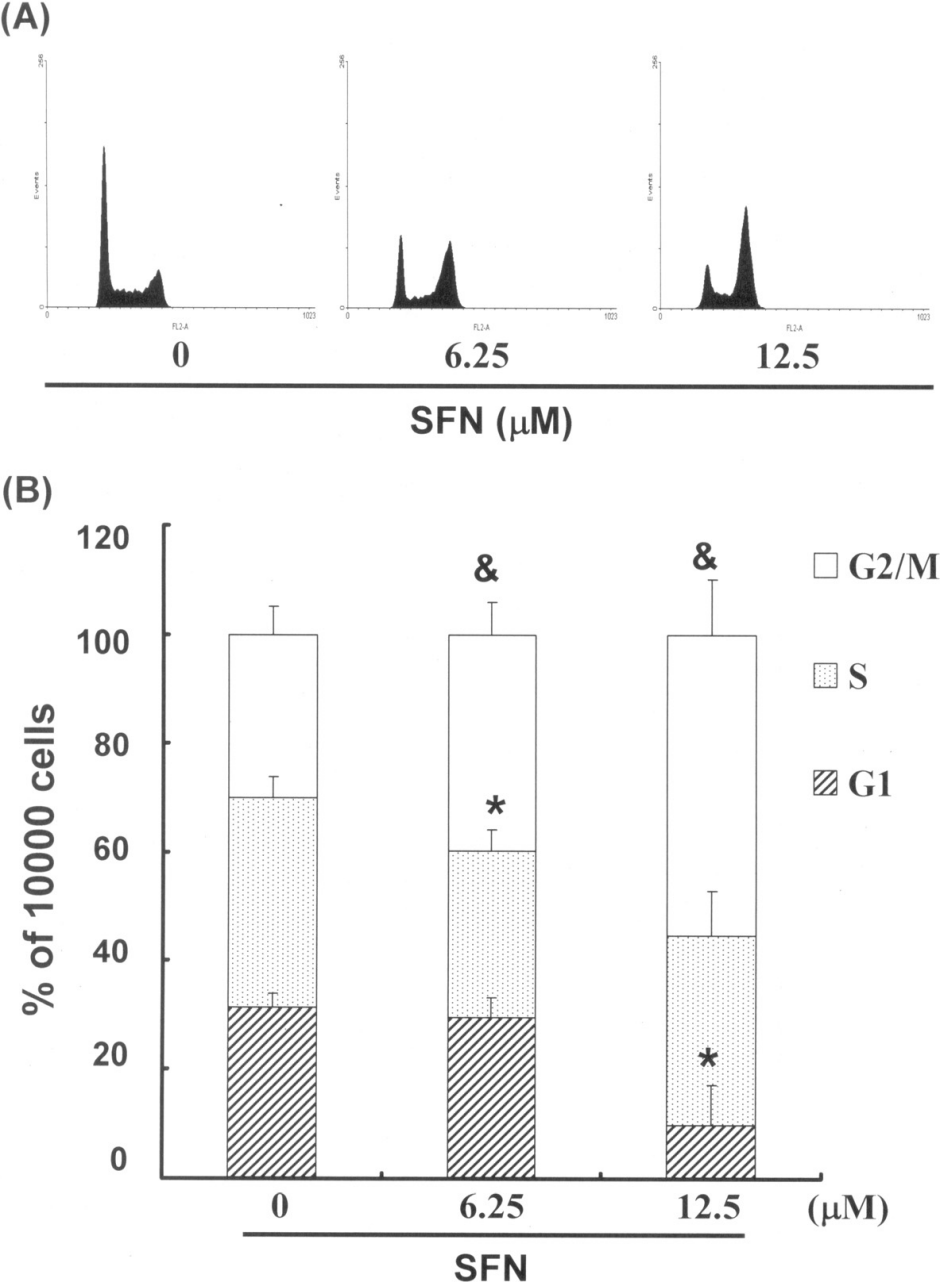
**Influence of SFN on cell cycle progression/distribution in PA-1 cells: (A) Cell cycle analysis of PA-1 cells after being cultured with SFN for 24 h; (B) SFN induced an increase in the number of G**_**2**_**/M phase cells (%).** The symbols * and & in each group of bars indicates that the differences resulting from treatment with SFN is statistically significant at P < 0.05.

The cell-cycle distribution of SFN-treated PA-1 cells was analyzed using flow cytometry. Prior to processing and analysis, cells were exposed to SFN for a total of 24 hours. As shown in Figure 2A, the cells exposed to SFN showed an increase in the number of cells in the G_2_/M phase (& p < 0.05 vs SFN 0 μM), compared with the number of untreated cells. This could imply that the PA-1 cells had undergone cell cycle arrest. We also found that treatment with SFN simultaneously reduced the number of cells in the G_1_ phase (* p < 0.05 vs SFN 0 μM) (Figure 2B).

### Cell cycle arrest by SFN in PA-1 cells via CDC2 down regulation and dissociation of the cyclin B1/CDC2 complex

**Figure 3:**
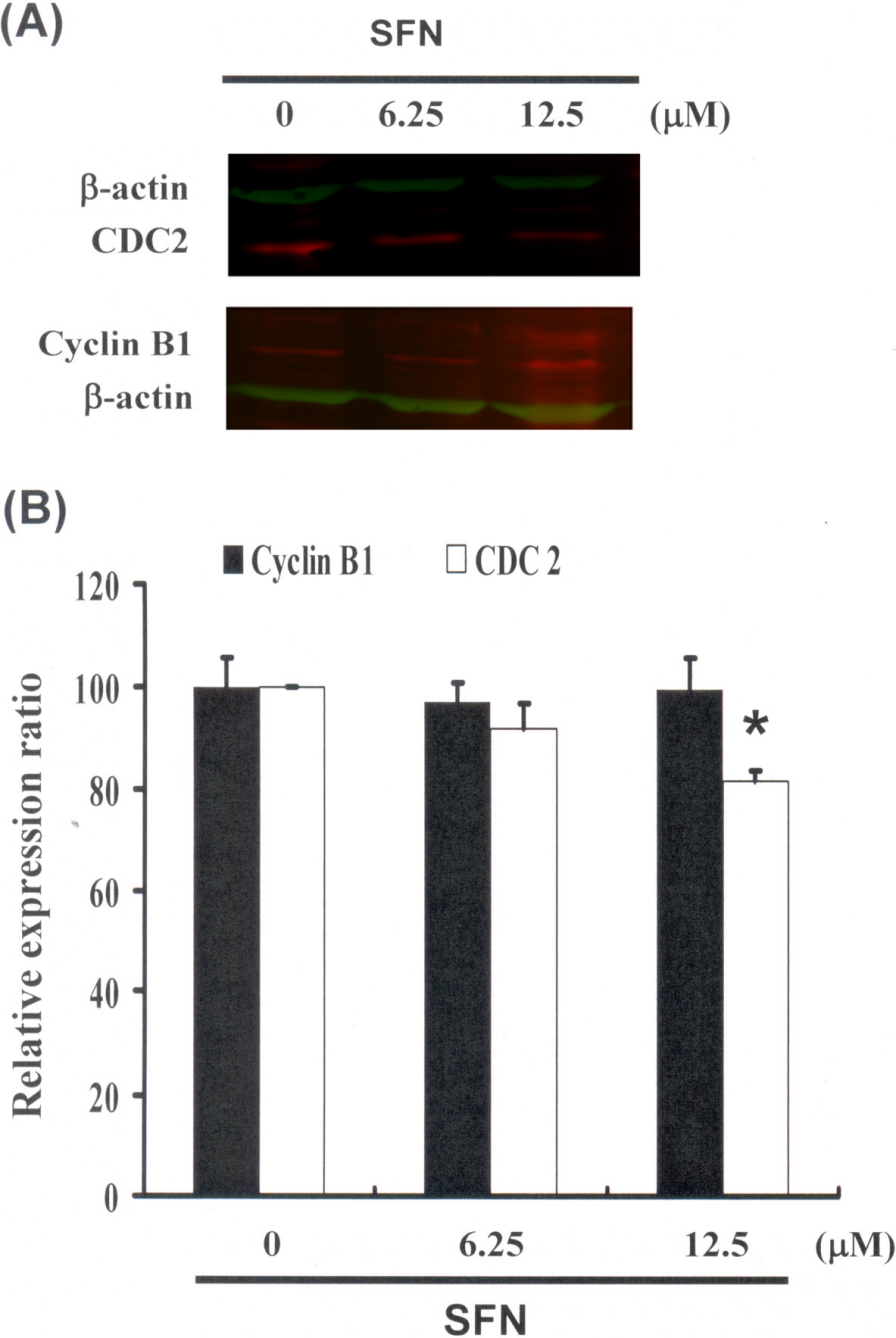
**Cell cycle arrest by SFN in PA-1 cells via inhibition of CDC2. Cells were treated with SFN followed by (A) western blot analysis (B) quantification of intensities by Li-COR near infrared imaging system.** Significant differences were determined at a level of *P < 0.05 versus the 0 μM control group.

**Figure 4:**
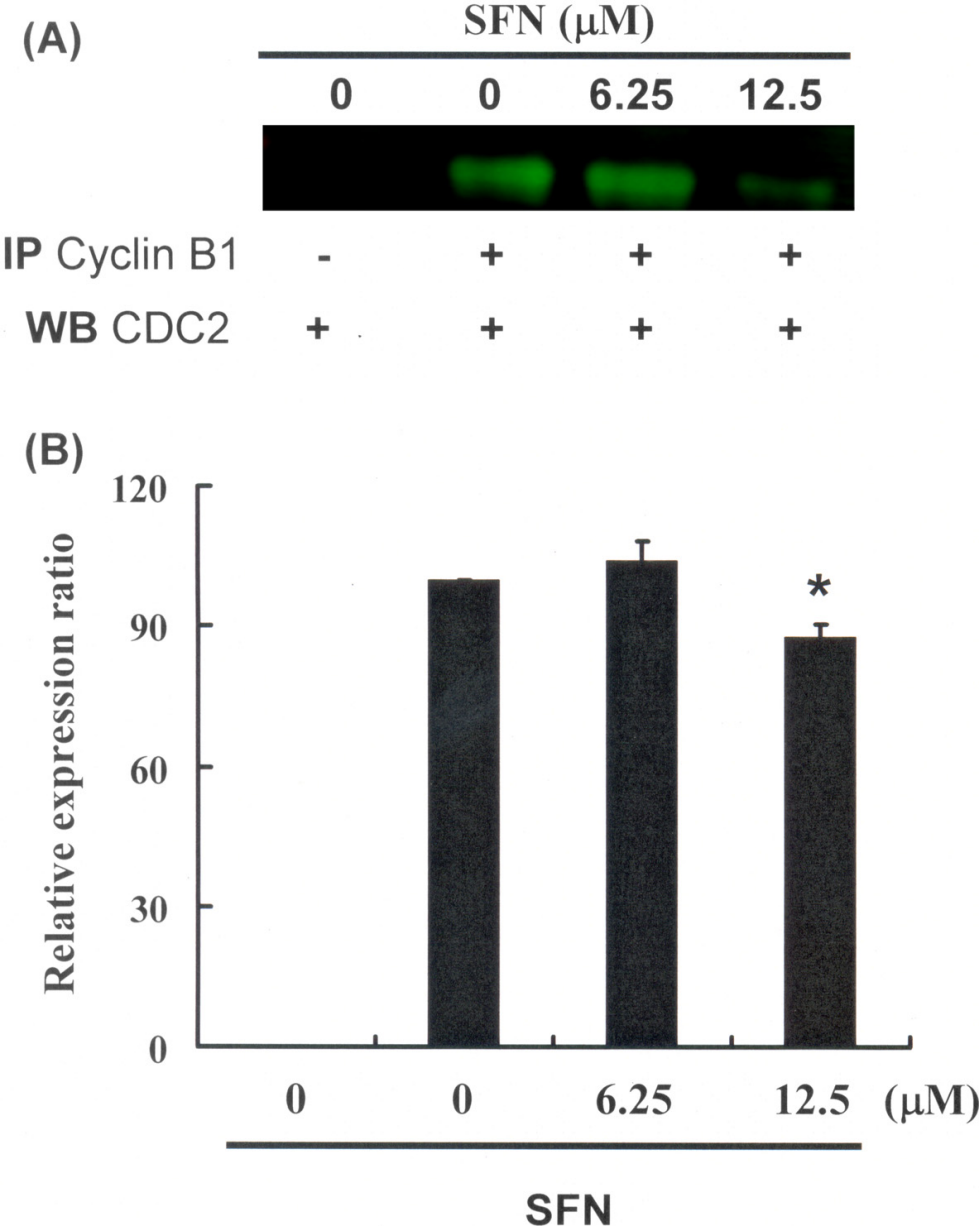
**Mitosis delay by SFN in PA-1 cells via inhibition of Cdc2-cyclin B1 complex dissociation: Cells were treated with SFN followed by (A) Co-IP and western blot analysis, and (B) quantification of intensities by Li-COR near infrared imaging system.** Significant differences were determined at a level of *P < 0.05 versus the 0 μM control group.

Figure 3 illustrates the immunoblotting of cellular proteins from PA-1 cells treated with SFN, revealing a decrease in CDC2 following incubation with SFN (Figure 3A). Cyclin B1 and CDC2 protein expression was quantified by measuring relative intensities. We found that CDC2 levels were significantly lower in cells incubated with SFN concentrations of 12.5 μM (Figure 3B). Moreover, the activity of the cyclin B1/CDC2 complex (important for G2-M transition during the cell cycle) was determined by Co-IP (Figure 4A) and quantified by measuring relative band intensities. We found that cyclin B1/CDC2 complex activity was significantly suppressed in cells incubated with an SFN concentration of 12.5 μM (Figure 4B).

These results indicate an increase of the cell population in G_2_/M phase via a down regulation of CDC2 and dissociation of the cyclin B1/CDC2 complex following incubation with SFN in PA-1 cells.

## Discussion

The results collected in this study using cell lines of human ovarian cancer provide experimental evidence indicating that SFN may induce irreversible cell cycle arrest during the G2/M phase. These dietary constituents demonstrate chemopreventive and chemotherapeutic potential through their ability to ameliorate the side effects of conventional chemotherapy [14].

This study investigated the in vitro expression of cyclin B1 and Cdc2 in PA-1 cells. The cells possess mechanisms to maintain genomic stability through cell cycle arrest [15]. At least two cell cycle checkpoints play a role in the cellular response, allowing the DNA to be repaired prior to DNA duplication (G1/S checkpoint) or mitosis (G2/M checkpoint) [16]. Cdc2 regulates mitosis and binds to cyclin B to form mitosis-promoting factor (MPF) [17]. The activity of MPF is regulated by the phosphorylation/dephosphorylation of Cdc2 as well as the accumulation of cyclin B protein and p53; GADD45 is also involved in a G2/M checkpoint and may participate in the regulation of Cdc2 kinase activity [18,19].

Other lines of evidence suggest that DNA damage excludes cyclin B1 from the nucleus, which promotes G2 arrest [20]. It is possible that cell cycle checkpoints delay cell cycle progression to allow additional time for the repair of DNA damage; however, our study found no direct evidence that DNA was repaired while the cells were arrested at the checkpoint.

Recent studies have shown that SFN inhibits the growth of tumor precursors and tumors in mice models when treatment is initiated at the time of carcinogen administration [10]. The co-inhibition of PI3K/AKT and ERK pathways activates FOXO transcription factor and enhances SFN-induced FOXO transcriptional activity, leading to cell cycle arrest and apoptosis [21].

## Conclusions

We conclude that G2 delay is a common response of tumor cells to chemotherapy with SFN. We further propose that mechanisms of this delay may be reduced expression of CDC2 and dissociation of the cyclin B1/CDC2 complex. Therefore, although certain chemopreventive effects of SFN and related isothiocyanate compounds have already been established with regard to ovarian cancer cells, SFN should be investigated further to confirm the additional antitumor properties proposed by our study.

## Competing interests

The authors declare that they have no competing interests.

## Authors’ contributions

CCC participated in its design and coordination and drafted the manuscript. YRY carried out the FACS analysis, cell culture and carried out the molecular studies. CMH performed the statistical analysis. YCH conceived of the study, and participated in its design and coordination and helped to draft the manuscript. All authors read and approved the final manuscript.
